# Come-back of phenanthridine and phenanthridinium derivatives in the 21st century

**DOI:** 10.3762/bjoc.10.312

**Published:** 2014-12-10

**Authors:** Lidija-Marija Tumir, Marijana Radić Stojković, Ivo Piantanida

**Affiliations:** 1 Laboratory for Study of Interactions of Biomacromolecules, Division of Organic Chemistry and Biochemistry, Ruđer Bošković Institute, Bijenička cesta 54, PO Box 180, HR-10002 Zagreb, Croatia

**Keywords:** ds-DNA and ds-RNA binding, intercalation, minor groove binding, nucleic acids, organic synthesis, phenanthridine, phenanthridinium

## Abstract

Phenanthridine derivatives are one of the most intensively studied families of biologically active compounds with efficient DNA binding capability. Attracting attention since DNA structure discovery (1960s), they were early recognized as a symbol of DNA intercalative binding, for many decades applied as gold-standard DNA- and RNA-fluorescent markers (ethidium bromide), probes for cell viability (propidium iodide), but also “ill-famed” for various toxic (genotoxic) and mutagenic effects. After two decades of low interest, the discovery of phenanthridine alkaloids and new studies of antiparasitic/antitumor properties of phenanthridine derivatives resulted in the strong increase of the scientific interest about the turn of this century. Here are summarized phenanthridine-related advances in the 21st century (2000-present period) with emphasis on the supramolecular interactions and bioorganic chemistry, as well as novel or improved synthetic approaches.

## Introduction

The search for therapeutic agents of the phenanthridine type has increased when the outstanding trypanocidal activity of some phenanthridinium compounds became apparent [1]. One of the most studied and used phenanthridine derivatives is 3,8-diamino-5-ethyl-6-phenylphenanthridinium known as ethidium bromide (EB), for many decades applied as gold-standard DNA- and RNA-fluorescent marker, and its close analogue (propidium iodide) as a probe for cell viability. Besides, an antiparasitic activity for EB was reported and it possesses significant antitumor activity [2–5] both in vivo and in vitro. Nevertheless, phenanthridine derivatives were rather neglected regarding their human medicinal applications due to potential carcinogenic and mutagenic properties of some derivatives (EB and analogues), which had negative influence on biomedically-oriented studies of the complete phenanthridine class till the end of the 20th century.

However, discovery of phenanthridine alkaloid analogues and in parallel new studies of antiparasitic properties of phenanthridine derivatives resulted in a strong increase of the scientific interest about the turn of this century, consequently yielding many publications at high impact chemical and biomedicinal journals, and patents covering various chemical, biochemical and biomedical uses. These results are up to our knowledge not summarized in any review within the last 10 years. Thus, taking advantage of our 20-year experience on phenanthridine derivatives (including very scarcely studied 4,9-diazapyrenium analogues with highly interesting biological effects), we summarized literature data (advances from 2000 to present) concerning supramolecular, bioorganic and medicinal chemistry, as well as novel or improved synthetic approaches.

## Review

### How to get phenanthridine: advances in synthetic pathways

**Scheme 1:**
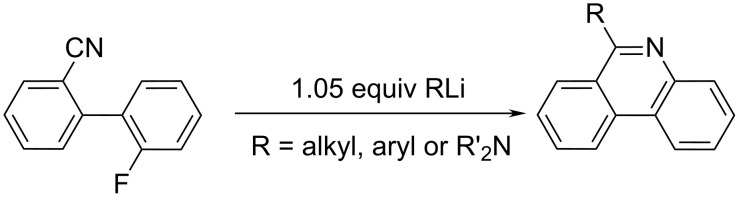
The Grignard-based synthesis of 6-alkyl phenanthridine.

Phenanthridine was first synthesized at the end of 19th century by Pictet and Ankersmit through pyrolysis of the condensation product of benzaldehyde and aniline [6]. The reaction conditions were improved by Morgan and Walls, based on a reaction including a cyclization of phenanthridine by dehydrative ring-closure with phosphorus oxychloride in boiling nitrobenzene [7]. Over the 20th century this method of phenanthridine preparation became the most common one. However, increased interest over the last decades resulted in many new and substantially different ways of phenanthridine synthesis with several different goals: to improve the reaction yield and to equip the phenanthridine moiety with various substituents, which were nicely summarized by Keller a decade ago [8]. We tried to survey the wide range of synthetic methods applied from 2000 on organizing them by similarity of reactants/catalysts or organic reactions; for instance the anionic ring-closure reactions using Grignard reagents (Scheme 1) [9], Bischler–Napieralski reactions [10], reduction of phenanthridones [11–12], free radical methodology, palladium/rhodium/iron-catalysed reactions, etc.

**Scheme 2:**
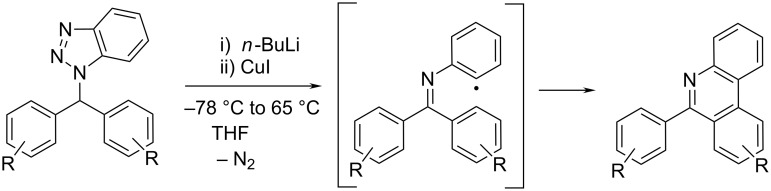
Radical-mediated synthesis of 6-arylphenanthridine [14].

One of the approaches to the large variety of 6-arylphenanthridine derivatives was the synthesis starting from benzotriazole derivatives of diarylmethanes, acridine, xanthene, thioxanthene, etc. It was based on the generation of a benzotriazole-stabilized carbanion followed by oxidation of this carbanion by copper iodide to form a radical. Subsequent elimination of nitrogen followed by ring closure yielded phenanthridine (Scheme 2) [13–14].

**Scheme 3:**
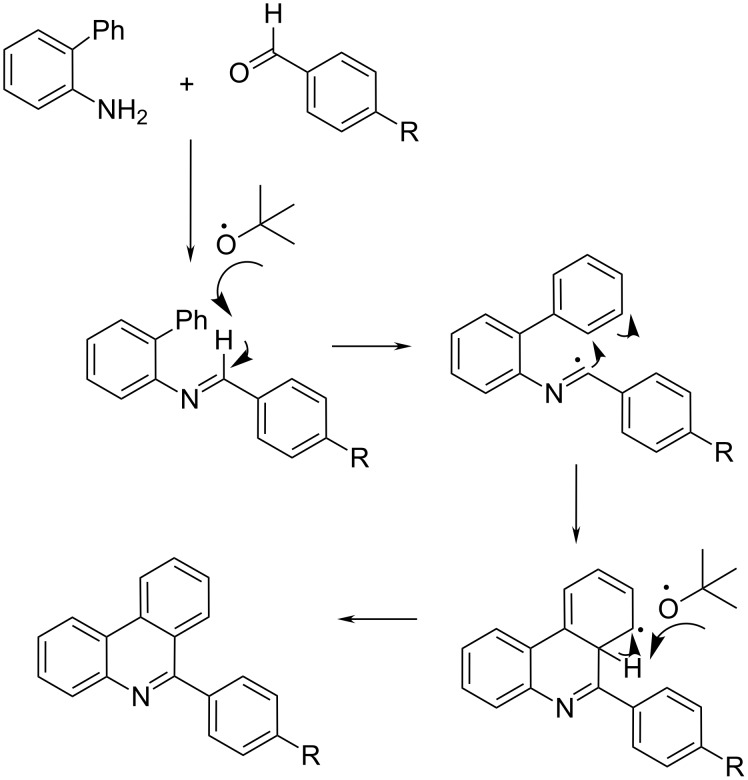
A *t*-BuO^•^ radical-assisted homolytic aromatic substitution mechanism proposed for the conversion of diarylimine into the 6-arylphenanthridine derivatives [16].

In the 1980s, Leardini et al. [15] have shown that under radical conditions via a homolytic aromatic substitution route diaryl-imines were suitable precursors to a number of 6-arylphenanthridine derivatives. The reaction proceeded by initial imidoyl-H atom abstraction by the electrophilic iPrO^•^ radical, and subsequently the intermediate underwent intramolecular cyclization and oxidative aromatization to form the phenanthridine ring. Bowman et al. [16] modified this route for safety reasons by application of di(*tert*-butyl)peroxide as a source of the *t*-BuO^•^ radical (Scheme 3). The required arylimines were prepared from aminobiphenyl and arylaldehyde in dichloromethane in the presence of molecular sieves at room temperature. Radical cyclisation in the presence of (*tert*-butyl)peroxide in chlorobenzene at 140–150 °C for 48 h, yielded the corresponding phenanthridines in moderate yields. The *t*-BuO^•^ radical eliminated the imine-H and formed the imidoyl radical, added to the phenyl ring. The homolytic aromatic substitution was terminated by H-atom abstraction by another *t*-BuO^•^ radical.

**Scheme 4:**
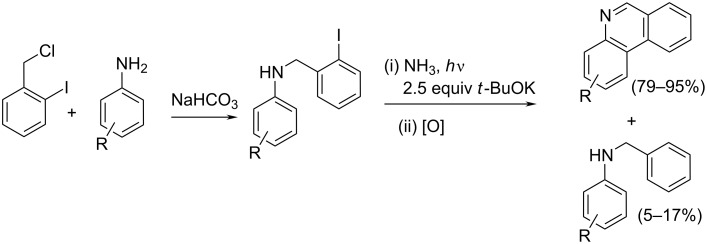
Synthesis of 5,6-unsubstituted phenanthridine starting from 2-iodobenzyl chloride and aniline [17].

Among very few routes targeting the synthesis of 5,6-unsubstituted phenanthridines, the here presented radical-based pathway used readily available *N*-(*o*-halobenzyl)arylamines as starting materials [17]. The *o*-haloarylbenzylamines (obtained by nucleophilic substitution of various anilines with 2-iodobenzyl chloride) gave the corresponding amide anions by an S_RN_1 substitution reaction in NH_3_ or DMSO as solvent under photoinitiation in the presence of excess *t*-BuOK. The photoinduced ET to the amide-anion resulted in its radical anion. After fragmentation of the C–I bond, an intramolecular cyclization occurred, and after acidification of the reaction medium, the oxidized phenanthridine compounds were obtained in very good yields (up to 95%, Scheme 4).

**Scheme 5:**
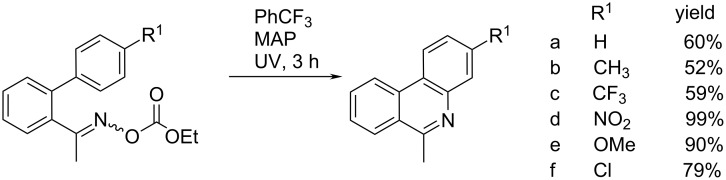
Phenanthridine synthesis initiated by UV-light irradiation photolysis of acetophenone *O*-ethoxycarbonyloxime derivatives at room temperature [18].

McBurney et al. prepared various N-heterocycles, using oxime carbonates as excellent precursors for the photoinduced generation of iminyl radicals, whereby at standard photolysis conditions, 3-substituted 6-methylphenanthridines were obtained in good to quantitative yields (52–99%, Scheme 5). Important advantages of the method are environmentally friendly and easily removable byproducts (CO_2_ and ethanol or phenol), and the negligible impact of the electronic nature of the substituent on the reaction [18].

**Scheme 6:**
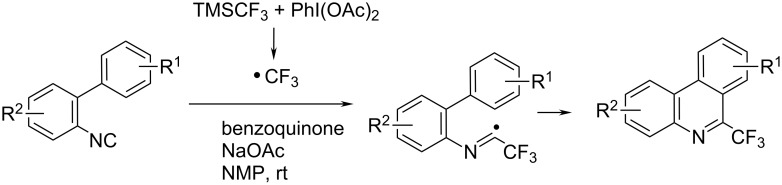
PhI(OAc)_2_-mediated oxidative cyclization of 2-isocyanobiphenyls with CF_3_SiMe_3_ [19–20].

The oxidative PhI(OAc)_2_-mediated cyclization of 2-isocyanobiphenyls with CF_3_SiMe_3_ under metal-free conditions showed to be a mild and efficient approach to 6-(trifluoromethyl)phenanthridines, characterised by good yields with high regioselectivity at ambient temperature (Scheme 6) [19–20].

**Scheme 7:**
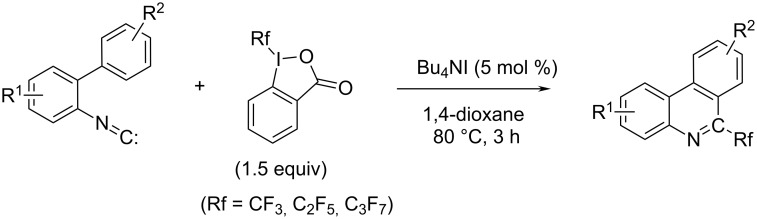
Targeting 6-perfluoroalkylphenanthridines [21–22].

Another radical-based route (targeting 6-perfluoroalkylphenanthridines), in which the transition metal is omitted, relied on the trifluoromethylation of isonitriles to yield trifluoromethylphenanthridines (Scheme 7) [21]. This approach employed the Togni reagent, and Bu_4_NI was applied as radical initiator; whereby phenanthridines were prepared in good to excellent yields [22]. Starting from the similar isonitrile structure, 6-aroylated phenanthridines via base promoted homolytic aromatic substitution (BHAS) can be prepared [23].

**Scheme 8:**
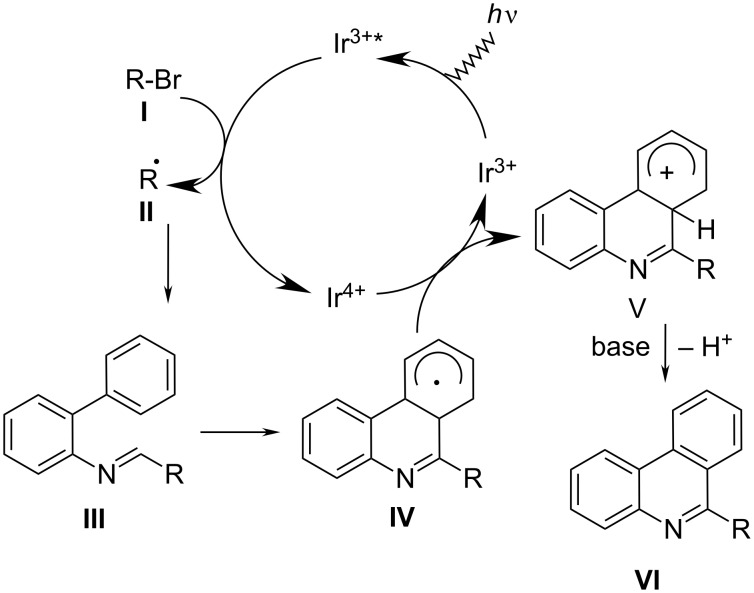
Easily accessible biphenyl isocyanides reacting under mild conditions (room temp., visible light irradiation, blue LED light source, N_2_, DMF, 10 h) with various common alkyl bromides by application the two-role catalyst [fac-Ir(ppy)_3_], gave phenanthridines in good yields [25].

Several photoinduced synthetic procedures were also applied. For instance, the photochemical cyclization of *N*-benzylanilines was used for asymmetrically substituted derivatives at phenanthridine side-rings and unsubstituted central ring [24]. The recently reported photo-conversion of various isocyanide biphenyls into alkylated phenanthridine derivatives under rather mild reaction conditions introduced several novelties (Scheme 8) [25]. The most intriguing is the double role of the photocatalyst [fac-Ir(ppy)_3_], consisting of photo-induced generation of alkyl radical **II** and oxidation of radical **IV** to cationic intermediate **V**, the latter process also regenerated the catalyst. Finally, the deprotonation assisted by base resulted in various 6-alkylated phenanthridines in excellent yields (>92%). The radical inhibitor 2,2,6,6-tetramethylpiperidin-1-oxyl (TEMPO) was applied to stop the transformation by a single electron transfer process.

**Scheme 9:**
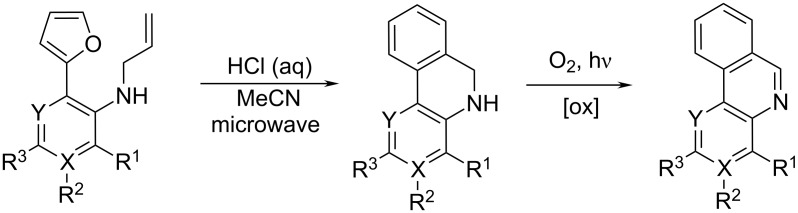
Microwave irradiation of Diels–Alder adduct followed by UV irradiation of dihydrophenanthridines yielded phenanthridines [26].

Intriguing combination of irradiation techniques (combined microwave-assisted and photochemical) offered a new route toward phenanthridines. Microwave-mediated intramolecular Diels–Alder cyclization of *o*-furyl(allylamino)arenes followed by spontaneous aromatization yielded dihydrophenanthridines, which upon exposure to UV light (315−400 nm) were oxidized into (aza)phenanthridines (Scheme 9) [26].

**Scheme 10:**
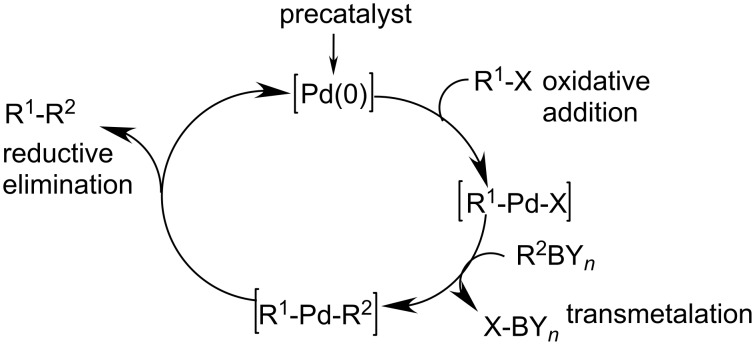
A representative palladium catalytic cycle.

Synthetic pathways based on the transition metal-catalysed functionalization of carbon–hydrogen (C–H) bonds and formations of C–C bonds are often used to access phenanthridines [27–29]. The most common are high-yield, palladium-based methodologies under mild conditions, due to their applicability on a large variety of aryl substituents [16] as well as potential for stereo and regioselectivity (Scheme 10) [30–31].

**Scheme 11:**
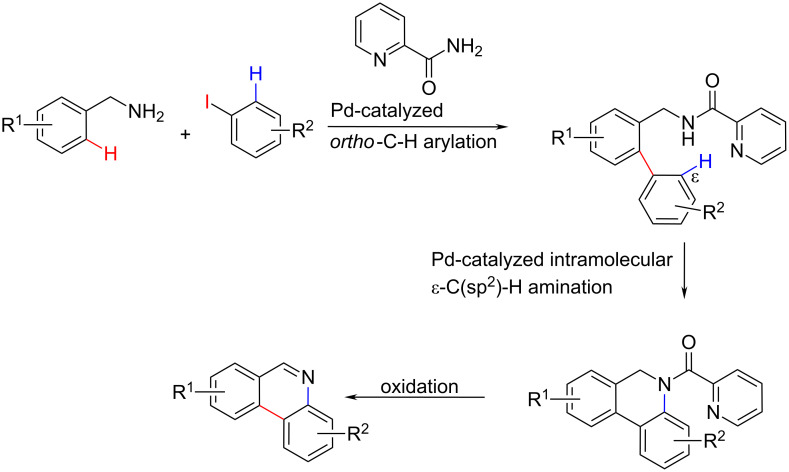
The common Pd-catalyst for the biphenyl conjugation results simultaneously in picolinamide-directed cyclisation; obtained *N*-picolinamide dihydrophenanthridine is easily converted to phenanthridine [32].

Among many examples, very recently a two-step phenanthridine synthesis stands out as a new strategy, characterised by two roles of the Pd-catalyst in the 1^st^ step, followed by simple and cost effective oxidation [32] (Scheme 11). This synthesis published by Pearson et al. was based on palladium-catalysed picolinamide-directed sequential C–H functionalization reactions, while readily available benzylamine and aryl iodide were used as precursors. In the first step the Pd-catalyzed reaction yielded a biaryl compound. The second step under the catalysis of Pd(OAc)_2_ comprised both cyclisation and oxidation in a single step: a dehydrogenative C–H amination with PhI(OAc)_2_ as oxidant and removal of the picolinamide group followed by oxidation with Cu(OAc)_2_. This strategy afforded phenanthridines in moderate to good yields (up to 65% for the second step).

**Scheme 12:**
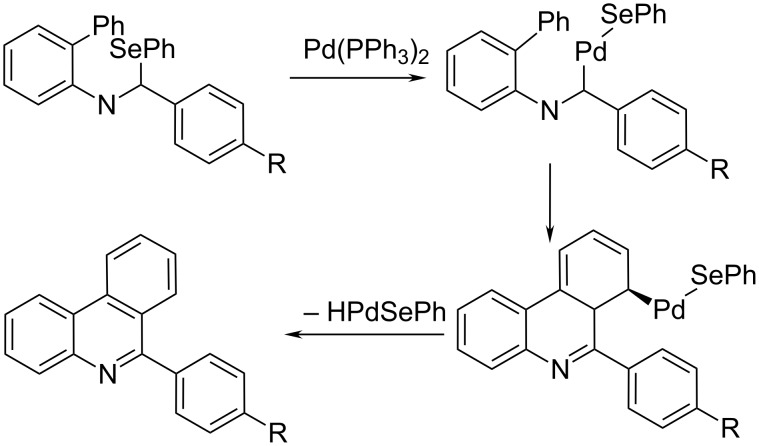
Pd(0)-mediated cyclisation of imidoyl-selenides forming 6-arylphenanthridine derivatives [16]. The insertion of the Pd(0) species into the carbon selenium bond followed by fast rearomatisation to phenanthridine is involved with the loss of HPdSePh.

Bowman et al. reported a palladium-mediated route using imidoyl-selenides as precursors besides the radical route. Comparison of the cyclisation yields for the same set of phenanthridine derivatives revealed an overall better efficiency of the *t*-BuO^•^ radical-assisted homolytic aromatic substitution of diarylimine (Scheme 3) in respect to the Pd(0)-mediated cyclisation of imidoyl-selenides (Scheme 12) [16]. Authors proposed insertion of a Pd(0) species into the carbon–selenium bond, followed by carbo-palladation onto the phenyl ring. This intermediate then undergoes rapid rearomatization with the loss of HPdSePh to give the phenanthridine.

**Scheme 13:**
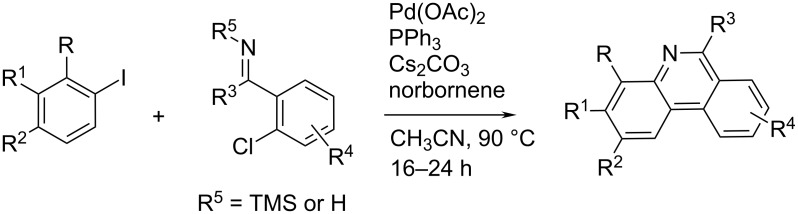
Palladium-catalysed phenanthridine synthesis.

Candito et al. reported a new and highly efficient method for the synthesis of variously substituted phenanthridine derivatives employing *N*-unsubstituted imines or *N*-silylimines [33]. The method is limited to *ortho*-substituted aryl iodides as starting material and also requires a convenient imine derivative allowing the cleavage of the nitrogen-attached group (R^5^) at some point in the catalytic cycle (Scheme 13). It is noteworthy that polar solvents (DMF, *N*-methylpyrrolidone, and acetonitrile) had favourable impact on the reaction.

**Scheme 14:**
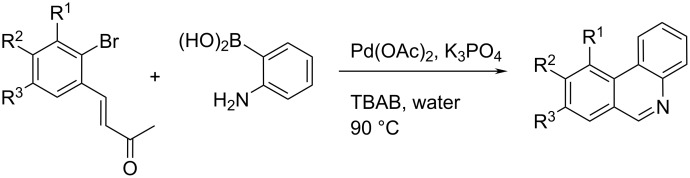
Aerobic domino Suzuki coupling combined with Michael addition reaction in the presence of a Pd(OAc)_2_/K_3_PO_4_ catalytic system in water [34–35].

Ghosh, Dhara et al. also reported a synthesis of substituted phenanthridines based on palladium-mediated Suzuki coupling (Scheme 14) [34–35]. Aerobic ligand-free domino Suzuki coupling–Michael addition reaction in the presence of Pd(OAc)_2_ and K_3_PO_4_ as a catalytic system in H_2_O was catalysed by palladium nanoparticles, that were generated in situ in water with the elimination of acetone.

**Scheme 15:**
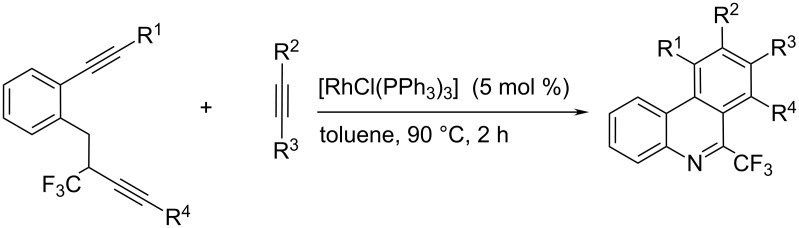
Rhodium-catalysed alkyne [2 + 2 + 2] cycloaddition reactions [36].

One of the major issues is the preparation of polysubstituted phenanthridines, in particular asymmetrically positioned on one of phenyl side-rings. An intriguing approach over rhodium-catalysed alkyne [2 + 2 + 2] cycloaddition reaction [36] (Scheme 15) offered a highly efficient method with excellent regioselectivity (in case of bulky groups), with additional advantage of the C-6 fluorinated methyl substituent – promising for PET probe development.

**Scheme 16:**
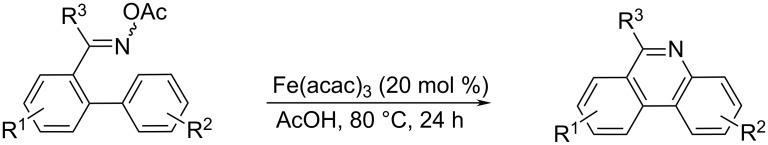
The *O*-acetyloximes derived from 2′-arylacetophenones underwent N–O bond cleavage and intramolecular *N*-arylation, followed by cross-coupling or directed C–H arylation [37].

Most of the metal catalysts employed for phenanthridine synthesis are rather expensive; therefore efforts were made to replace them with cheaper analogues. One successful approach included iron(III) acetylacetonate in acetic acid as catalytic agent (Scheme 16) [37], whereby *O*-acetyl oximes obtained from 2′-arylacetophenones underwent N–O bond cleavage and intramolecular *N*-arylation. The following conventional cross-coupling or directed C–H arylation resulted in substituted phenanthridines.

Homolytic aromatic substitution (HAS) by an aryl radical was used for the construction of biaryl motifs as alternative to transition metal-catalysed C–H bond arylation. That approach was also implemented in the two-component cyclization in the synthesis of phenanthridine derivatives [38]. The starting isocyanide biphenyl (similar to Scheme 8) reacts with the phenyl radical generated from phenylboronic acid and a manganese salt followed by spontaneous cyclisation and aromatisation.

**Scheme 17:**
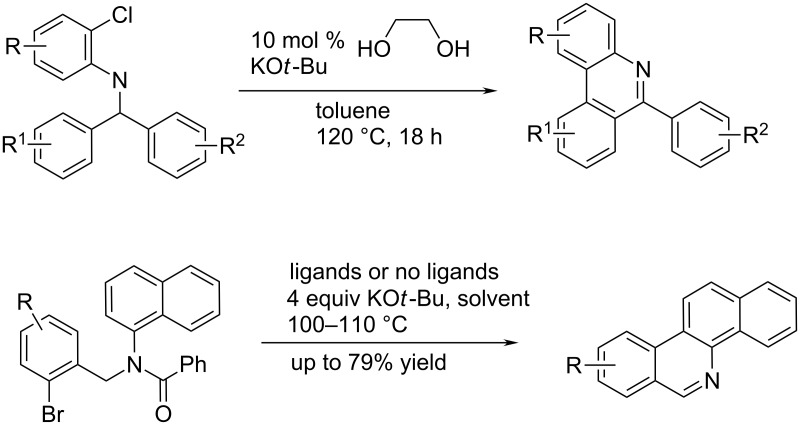
C–H arylation with aryl chloride in the presence of a simple diol complex with KO*t*-Bu (top) [39]; for some cases it worked also in the absence of diol (bottom) [40].

Trying to omit the expensive metal catalysts, several successful attempts of a transition metal-free approach for phenanthridine synthesis were reported. For instance application of a simple diol combined with KO*t*-Bu resulted in intramolecular C–H arylation to give the respective phenanthridine derivatives (Scheme 17 top) [39]. More recently, a similar procedure worked just in the presence of KO*t*-Bu by intramolecular homolytic aromatic substitution (HAS), without the use of an organic molecule as ligand to give benzo[*c*]phenanthridine derivatives (Scheme 17 bottom) [40].

**Scheme 18:**
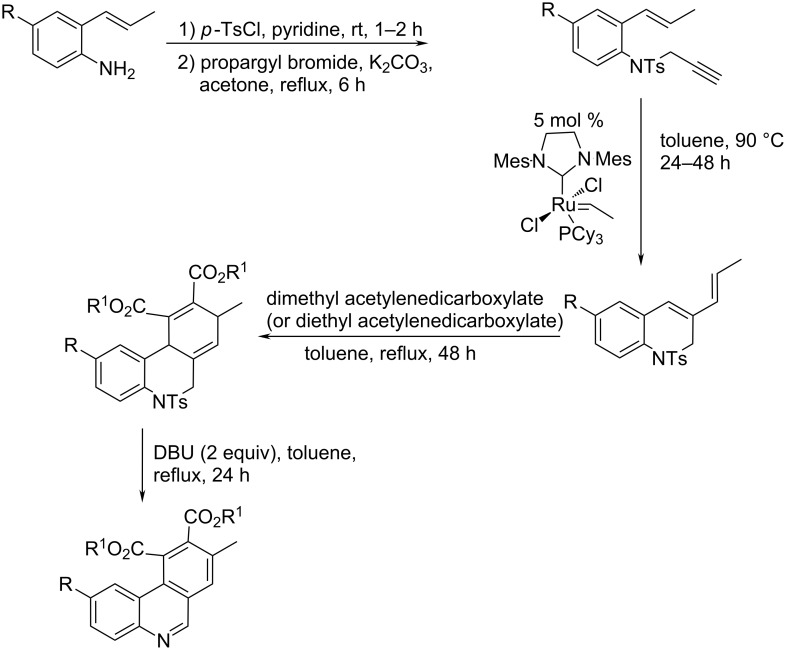
The subsequent aza-Claisen rearrangement, ring-closing enyne metathesis and Diels–Alder reaction – a new “three-atom economic process” of phenanthridine synthesis [41].

An unique approach to the phenanthridine core starting from a simple disubstituted aniline relied on the aza-Claisen rearrangement, ring-closing enyne metathesis and Diels–Alder reaction [41] (Scheme 18). The obtained phenanthridine derivatives were polysubstituted at the phenyl side-rings, while retaining the unsubstituted central heterocyclic double bond. The diversity of the aza-Claisen rearrangement allows the application of this approach to other related heterocyclic systems.

**Scheme 19:**
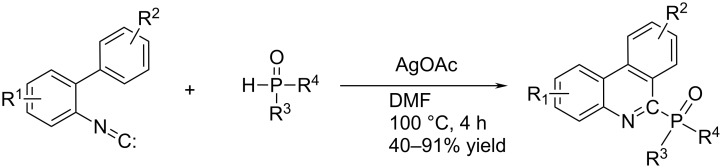
Phenanthridine central-ring cyclisation with simultaneous radical-driven phosphorylation [42].

The preparation of a new variety of analogues, namely 6-phosphorylated phenanthridines was very recently reported, whereby central-ring cyclisation was accompanied with simultaneous phosphorylation [42] (Scheme 19). The particular importance of this economic and highly efficient synthetic method is the complementarity of the starting material, the easy availability of 2-isocyanobiphenyls, which could be converted to variously substituted phenanthridines in several ways (Scheme 7 and Scheme 8).

**Scheme 20:**
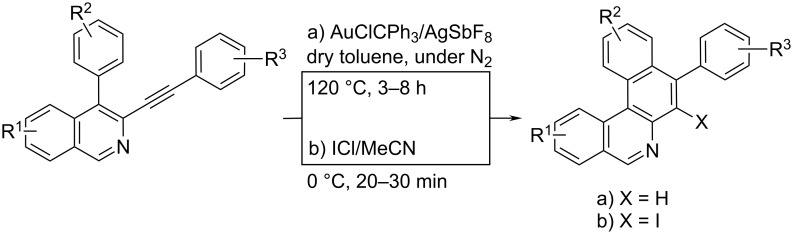
Three component reaction yielding the benzo[*a*]phenanthridine core in excellent yields [44].

Because of the recent strong focus on benzophenanthridines due to their potent antitumor and antiinfectious activities [43], we have chosen one recent synthetic approach (differing from afore listed examples) to benzo[*a*]phenanthridines as close analogues of phenanthridine (Scheme 20) [44]. The synthesis by multicomponent tandem reaction/carbocyclization starts with the formation of a 4-aryl-3-arylethynylisoquinoline from 2-bromobenzaldehyde/*tert*-butylamine/1,3-diyne. The second (in situ) step is based on the ring closure, either via gold/silver-catalysed intramolecular hydroarylation or via iodo-catalysed regioselective 6-endo-dig electrophilic cyclization.

**Scheme 21:**
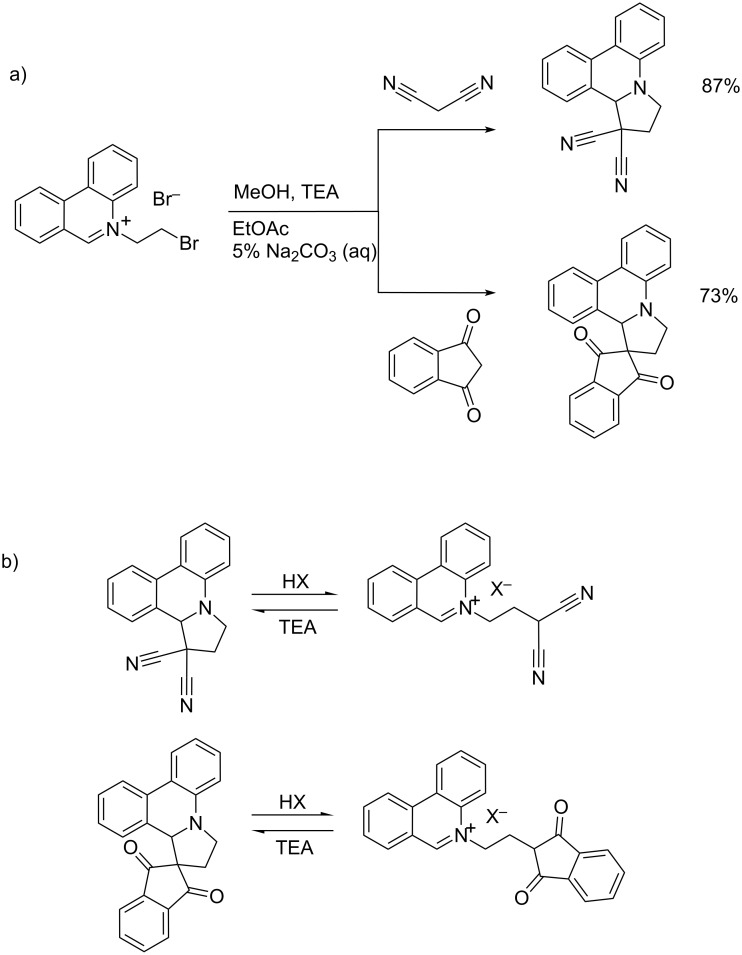
a) Reaction of malononitrile and 1,3-indandione with BEP to form the cyclised DPP products; b) pH controlled reversible cyclisation process of DPP compounds [45].

Kitson et al. synthesized a class of 2,3-dihydro-12*H*-pyrrolo[1,2-*f*]phenanthridine (DPP) derivatives starting from malononitrile and 1,3-indandione as the initial nucleophiles, which reacted with *N*-bromoethylphenanthridinium bromide to give DPP-dicarbonitrile and DPP-indandione, respectively. Particularly an interesting property of these DPP products is the reversible, pH controlled ring-opening-cyclisation process, whereby at acidic conditions DPP undergoes rearomatisation of the phenanthridinium ring system (Scheme 21a), which at basic conditions (TEA) switches back to the initial DPP structure (Scheme 21b) [45].

### Summary of synthetic advances

Among many given examples of the phenanthridine synthesis, currently two most common routes are the synthesis under radical conditions [12–24] and the synthesis based on transition metal-catalysis [15,27–37].

The main advantage of the radical-based phenanthridine synthesis is easy available and generally cheap starting material (benzotriazole, aminobiphenyl, arylaldehyde, *N*-(*ortho*-halobenzyl)arylamines, oxime carbonates, isocyanobiphenyls, etc.). Phenanthridines are usually obtained within 2–3 reaction steps, by application of different radical initiators. An intriguing alternative is the radical generation by UV irradiation with or even without a photocatalyst. The major advantage of radical-based routes are usually mild reaction conditions, while reaction yields, after optimization of the synthesis parameters, span from 50–90%, mostly depending on the substituents attached to the starting material. The radical-based synthesis is typically used for the preparation 6-aryl or 6-alkylphenanthridine derivatives and 6-phosphorylated analogues, equipped with one or two additional substituents, usually positioned on the phenanthridine positions C1–4 or position C8.

Similarly to the radical-based synthesis, a synthetic approach based on transition metal-catalysis also allows the phenanthridine preparation from easily available starting material (benzylamine, aryl iodide, imines, etc.) in few reaction steps, under mild reaction conditions and with yields within the 50–90% range. The great advantage of this approach is the very broad versatility in preparation of phenanthridine derivatives, poly-substituted on the phenyl side-rings by a large variety of substituents, as well as stereo- and regioselectivity (particularly for the bulky groups). Nevertheless, due to the most common metal catalyst (palladium) this method is significantly more expensive and less environmentally friendly than radical-based methods. To address these disadvantages, in the last decade particular attention was given to the replacement of the expensive palladium catalyst, for instance by iron [37]. However, major impact was made by introduction of the cheap and environmentally friendly intramolecular homolytic aromatic substitution (HAS) reaction with the aid of the organo-catalysis; although it is currently applicable for the preparation of only a limited variety of phenanthridine derivatives and benzophenanthridines but future prospects are very promising.

Aside two most common ways to prepare the phenanthridine moiety, here are described several innovative approaches, with potential to be developed for a large versatility of phenanthridine derivatives or application of previously not used starting materials (for instance microwave-mediated intramolecular Diels−Alder cyclization of *o*-furyl(allylamino)arenes).

For the most of DNA or RNA targeted applications the phenanthridine is converted to the positively charged phenanthridinium cation by simple alkylation of the phenanthridine heterocyclic N5 nitrogen (thus giving permanent positive charge) or by the N5 nitrogen protonation at weakly acidic conditions (p*K*(N5) ca. 5.5–6) yielding reversible positive charge. Here are also described novel approaches to reversible positively charged (DPP and DIP derivatives [45]), which are related to remarkable structural features of the naturally occurring benzophenanthridine alkaloids – pH-dependent structural transition between the iminium (positively charged) and alkanolamine (neutral) form [46].

### Structural features of phenanthridines and phenanthridinium cations related to DNA and RNA binding

**Figure 1:**
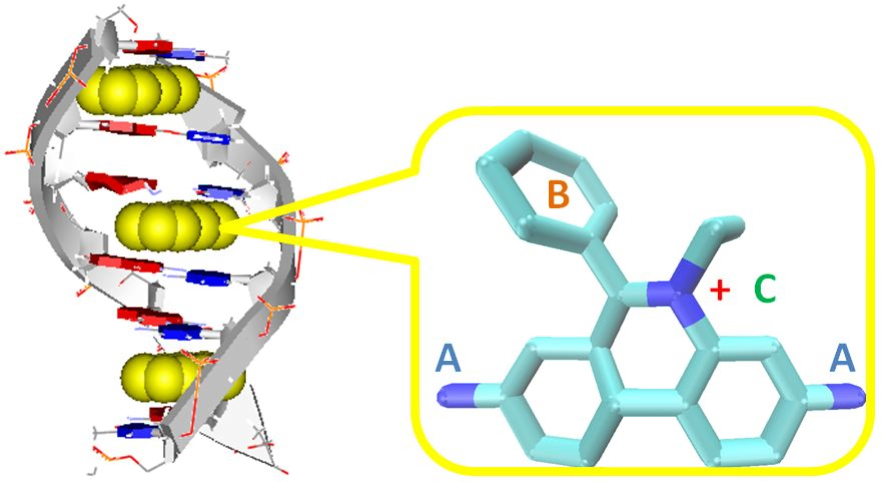
Schematic presentation of the intercalative binding mode by the neighbour exclusion principle and important structural features of ethidium bromide: A**)** amino substituents responsible for fluorescence increase upon DNA intercalation; B) phenyl substituent for steric control and also impact on fluorimetric properties; C) permanent positive charge for aqueous solubility and electrostatic attraction to the DNA or RNA phosphate backbone.

Structural studies on the phenanthridine system were mostly driven by its most widespread use as DNA and RNA intercalator (Figure 1) and/or fluorescent marker (ethidium bromide/propidium iodide) for ds-DNA and ds-RNA [47]. The phenanthridine structural features incorporate a unique set of properties related to the interaction with DNA and RNA (Figure 1): size and curvature of the aromatic surface corresponds to the basepair shape, whereas the high polarizability (and permanent positive charge of N-5 alkylated derivatives) also plays an important role in aromatic and electrostatic interactions with polynucleotides. Moreover, non-covalent interactions with DNA and RNA can be reversibly controlled by a pH-induced positive charge at the heterocyclic nitrogen N-5, and strong electron affinity and polar groups at the 3 and/or 8 position of the phenanthridine can efficiently and predictably regulate the spectroscopic response (UV–vis and fluorescence) of the chromophore [48].

The understanding of the intercalation process requires a detailed knowledge of the energetics, thermodynamics and structural equilibrium – surprisingly few studies endeavoured to determine important parameters for such classical intercalator as ethidium bromide [49]. The most recent and very extensive theoretical study compared positively charged ethidium bromide and its neutral analogue, revealing detailed description of the forces included in the intercalation process, stressing the dispersion energy as a control factor [50]. Moreover, a number of kinetic measurements provided for the binding of ligands to DNA additionally clarify mechanistic details that are not apparent from equilibrium measurements [51].

Another, very comprehensive approach, relying mostly on the experimental data of X-ray crystallography, UV–vis, fluorescence and NMR spectroscopy, determined that the fine interplay between electron donating and electron withdrawing effects mediated by its nitrogen atoms defines the spectroscopic properties of ethidium bromide (EB) and its derivatives [48]. It turned out that, despite the positive charge of ethidium bromide, most of ethidium's aromatic carbon and hydrogen atoms have high electron densities compared to the 6-phenylphenanthridines. Thus, the electron-donor properties of the exocyclic amines, especially at 8-position have a stronger influence on the electron density of aromatic atoms than the electron withdrawing effects of endocyclic iminium. Fine tuning of electron properties of EB can be easily achieved via chemical modulation of its amino groups at 3 and 8 positions of the phenanthridine ring [52–53]. Systematic changing of the ethidium bromide exocyclic amines into guanidine, pyrrole, urea, and various substituted ureas revealed importance of electron-donor properties of substituents at the 3- and 8-position of the phenanthridinium relative to the unmodified primary amino groups. Namely, derivatives of EB having substituents with weaker electron-donor properties exhibited a stronger fluorescence emission than EB, while a stronger electron-donating substituent exhibited a much lower fluorescence emission. Such behaviour could be attributed to the ethidium exocyclic amines enabling by electron donation a non-radiative decay of phenanthridinium excited state, rather more likely than the previously proposed mechanism of water-induced deprotonation of phenanthridinium exocyclic amines, causing excited chromophore fluorescence quenching [54–55].

Taking into account the research results of several other groups, a general rule could be drawn that phenanthridines with no amino groups yield strong fluorescence in water but emission is totally quenched by DNA binding; one amino group at (usually) position 8 results in only a small fluorescence change in the complex with DNA, while two amino groups in 3,8-position result in a weak fluorescence with strong emission increase upon DNA binding [52–53,56].

A pronounced influence of the substituent at phenanthridine position 6 on the optical properties of the chromophore also had significant impact on the binding affinity toward ds-DNA. The comparison of three substituents in 6-position, 4-*N*,*N*-diethylaminophenyl, phenyl (EB) and methyl, revealed that the first one exhibits the strongest DNA binding affinity and the strongest fluorescence enhancement. That was related to the twist angle in the excited state between the 6-phenyl ring and the phenanthridinium chromophore, which controls the non-radiative relaxation [56].

### Substituted phenanthridine derivatives

**Figure 2:**
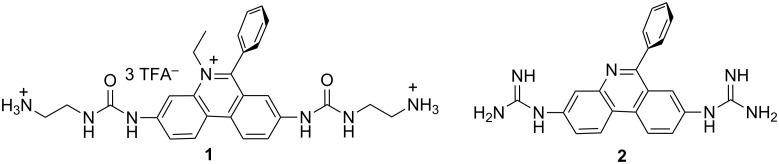
Urea and guanidine derivatives of EB with modified DNA interactions [57].

In particular guanidine- and urea-substituted derivatives attracted a lot of attention due to the different interactions with various DNA and RNA. The ability of ethidium bromide analogues to inhibit the HIV-1 Rev–Rev Response Element (RRE) interaction, as well as their affinity to calf thymus (ct)DNA was analysed. One derivative (Figure 2, **1**) displayed an enhanced affinity for HIV-1 RRE and a lower DNA affinity (i.e., lower mutagenic activities) compared with ethidium bromide. A recent study showed that substitution of both ethidium bromide (EB) and exocyclic amines by guanidines converted the classical intercalator (EB) into a DNA minor groove binder [57]. The most intriguingly, binding mode change did not weaken the DNA affinity, thus the affinity of guanidine derivative **2** (Figure 2) towards AT-rich DNA sequences was significantly stronger compared to ethidium and comparable to that of the known DNA minor groove binder furamidine.

**Figure 3:**
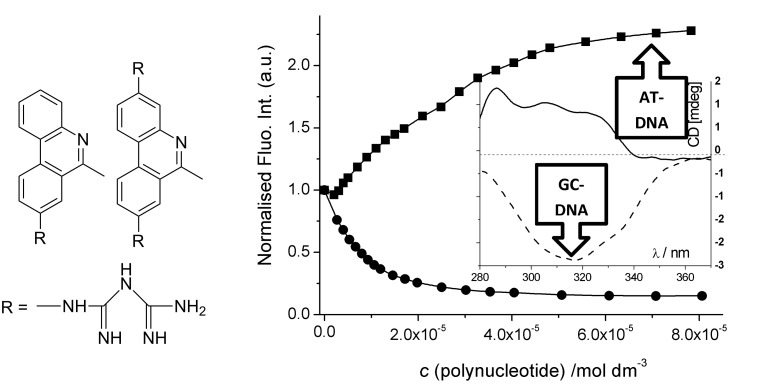
Structure of mono- (**3**) and bis-biguanide (**4**) derivative. Fluorescence (y-axis normalised to starting fluorescence of free **4,** *c* = 1.0 × 10^−6^ mol dm^−3^) was quenched by GC-DNA and increased for AT-DNA. Inset: induced (I)CD spectra λ > 280 nm of **4** (r(**4**)/DNA = 0.3; *c*(DNA) = 2 × 10^−5^ mol dm^−3^) – strong positive ICD band for AT-DNA and negative ICD band for GC-DNA. Adapted with permission from [58]. Copyright 2011 The Royal Society of Chemistry.

The above mentioned guanidine-induced switch of the DNA- and RNA-binding mode [57] inspired a design of derivatives equipped with biguanide groups at 3 and/or 8 positions [58] (Figure 3), under the presumption that the extended H-bond-rich system should increase the ability of the chromophore to differ among various shapes of ds-DNA- and ds-RNA-grooves. Both, mono- (**3**) and bis-biguanide (**4**), efficiently discriminate between dAdT and dGdC polynucleotides by opposite changes of compound fluorescence, as well as opposite induced (I)CD bands (Figure 3). Moreover, both, **3** and **4**, show the binding to AU-RNA by a different fluorimetric and CD response in respect to DNA-binding. Observed recognition between various DNA and RNA polynucleotides was attributed to the switch of the binding mode (intercalation into dGdC-DNA and AU-RNA and minor groove binding into dAdT-DNA).

A common strategy for the modification of DNA- and RNA-targeting molecules by preparation of homo-dimers was also implemented on the phenanthridine moiety – many ethidium bromide-based dimers were prepared and reviewed in the last two decades of the 20th century, thus here will be presented results from 2000 on.

**Scheme 22:**
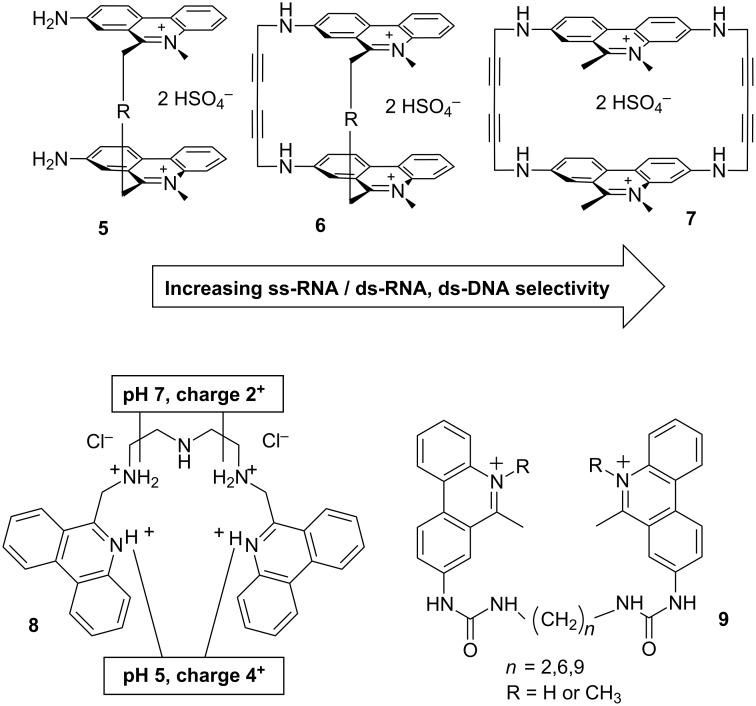
Bis-phenanthridinium derivatives (**5**–**7**; inert aliphatic linkers, R = –(CH_2_)_4_– or –(CH_2_)_6_–): rigidity of a “cage” – steric control of binding site. Triamine-linked bis-phenanthridine **8**, note reversible doubling of positive charges at pH 5 in respect to neutral conditions (pH 7). Bis-urea phenanthridines (general structure **9**): different from amino analogues (**5**) by fluorimetric response and DNA- and RNA-binding modes.

Systematic variation of steric and/or electrostatic effects by means of type, number, length and flexibility of linkers connecting two phenanthridine units is presented in Scheme 22.

The ability of switching on/off the charge of phenanthridine heterocyclic N5 via its protonation at weakly acidic pH (pK 5–6) was utilized in a design of phenanthridine derivatives to alter significantly their binding preferences toward polynucleotides. Among several examples, the most intriguing pH controlled binding of nucleotides and nucleic acids showed bis-phenanthridine triamine [59] (**8**, Scheme 22). Compound **8** intercalated with only one phenanthridinium subunit into all ds-DNA and ds-RNA, while additional interactions of the other subunit within the grooves finely tuned the recognition between various ds-polynucleotides. The sensitivity of spectroscopic response was particularly pronounced for ss-RNA, whereby at weakly acidic pH compound **8** exhibited specific fluorimetric sensing of poly(G) among other studied ss-polynucleotides.

Cyclic cage-like bis-phenanthridinium derivatives (Scheme 16; general structure **7**), with a rigid structure allowing accommodation of only one nucleobase, showed pronounced ss-RNA over ds-RNA/DNA selectivity [60], whereas more flexible cyclic (**6**) and acyclic analogues (**5**) [61] revealed opposite preference, stressing the importance of steric control over selectivity (Scheme 16). The selectivity of **7** was based on the switch of binding mode; the very rigid pocket between two phenanthridinium moieties allows only bis-intercalation into single-stranded polynucleotides and only binding with double-stranded polynucleotides in non-intercalative mode (most likely within the DNA and RNA grooves). Moreover, the cage-like binding pocket of bisphenanthridiniums **7** showed to be sensitive to the minor structural differences between mononucleotides, yielding a very selective fluorimetric response upon binding of AMP in respect to other nucleotides. In addition, the observed selectivity towards poly(G) and poly(A) can be beneficial in biological applications for instance to influence the mRNA-function via binding to the poly(A) tail [62–64] and inhibition of the HIV-1 replication by targeting recognition of the polypurine tract by reverse transcriptase [65].

In a series of N5-protonated urea-substituted bis-phenanthridinium derivatives (Scheme 22, general structure **9**), the variation of the linker length connecting two urea-phenanthridinium conjugates significantly influenced the efficiency of intramolecular interactions between two phenanthridinium subunits and consequently their DNA- and RNA-binding mode (shorter linker–minor groove binding, the longest linker–intercalation) [66–67]. In addition, the derivative with the longest linker was, to the best of our knowledge, the first bis-phenanthridine-based intercalator able to differentiate between A–U(T) and G–C base pairs by sign of opposite fluorimetric response. An introduction of the permanent positive charge by methylation of the heterocyclic nitrogen changed the binding mode of the conjugates with shorter linkers from minor groove binding to intercalation and also resulted in significantly higher biological potency in respect to non-methylated analogues [67]. Moreover, the observed DNA and RNA interactions were also distinctively different from previously studied aliphatic-linker analogues (**5**), pointing out the decisive role of urea-linker interactions.

**Figure 4:**
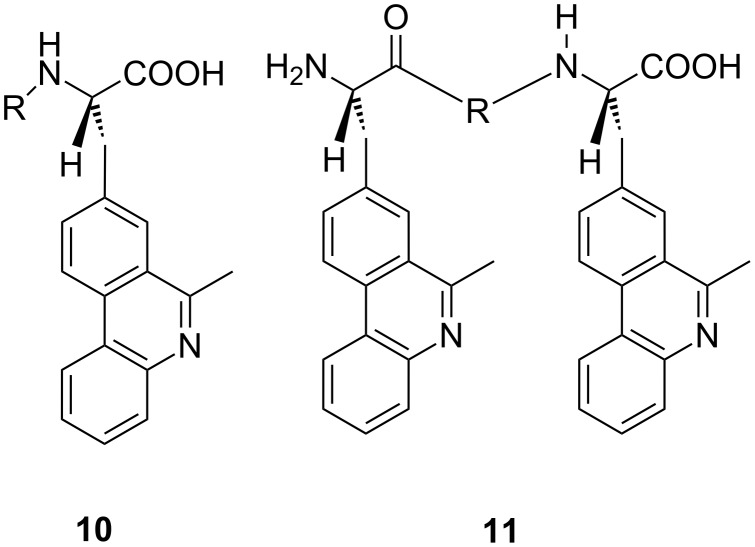
Series of amino acid–phenanthridine building blocks (general structure **10**; R = H; Gly) and peptide-bridged bis-phenanthridine derivatives (general structure **11**; R = X; Gly; Gly–Gly) [68].

The common approach to complex small molecules targeting DNA and RNA usually required a number of consecutive synthetic steps, which made modification of the interesting structures a laborious and time-consuming task, quite often being the bottle-neck in the structure–activity relation research. With aim to facilitate structural modifications in DNA and RNA targeting by oligo-aryl derivatives, new amino acids with phenanthridine attached to the side chain were prepared and the solid phase synthesis of novel peptide-bridged bis-phenanthridine derivatives was developed (Figure 4) [68], whereby the position of the DNA-active chromophore in the peptide backbone as well as the structural characteristics of the linker between them can easily be modified. In the first series of peptide-bridged bis-phenanthridine derivatives, derivative **11** with the shortest linker formed an intramolecular excimer, characterised by the specific fluorescence band sensitive to the pH as well as on the interactions with ds-DNA. Interestingly, all peptide-based phenanthridines revealed excellent water solubility combined with low in vitro toxicity, thus being good candidates for development of new safe fluorimetric DNA and RNA dyes.

**Figure 5:**
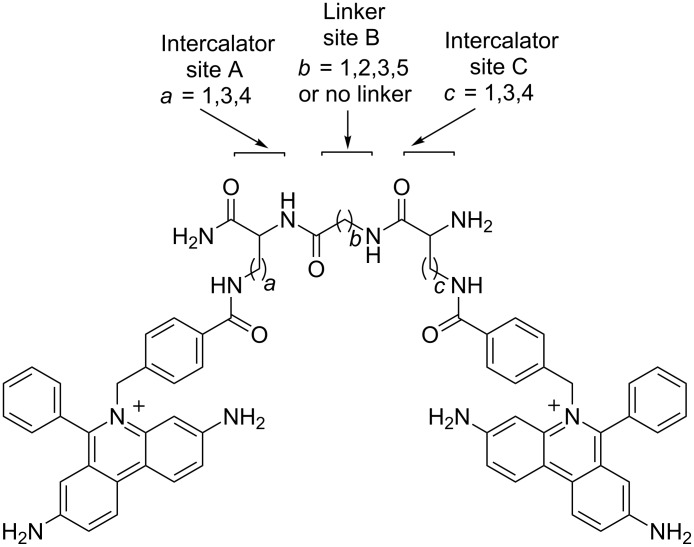
General structure of 45 bis-ethidium bromide analogues. Reproduced with permission from [69]. Copyright 2012 Elsevier Ltd.

Another large series of phenanthridinium-homodimers was constructed by linking two ethidium bromide subunits by peptide-like linkers of variable flexibility and rich in hydrogen-bonding possibilities within the DNA grooves (Figure 5). The resulting bis-intercalators (in comparison to the monomeric analogues) revealed significantly increased DNA-binding affinity and consequently enhanced telomerase and reverse transcriptase inhibition [69].

### Conjugates of phenanthridine with other DNA and RNA active moieties

Another common approach to increased selectivity of DNA- and RNA-targeting small molecules is the design of complex conjugates consisting of several DNA- and RNA-active parts (e.g., intercalator, groove binder, electrostatically binding component, various sterically directing parameters). The phenanthridine moiety was quite often used as presumably intercalating unit, although in some cases a switch of the binding site to the minor groove was reported.

**Scheme 23:**
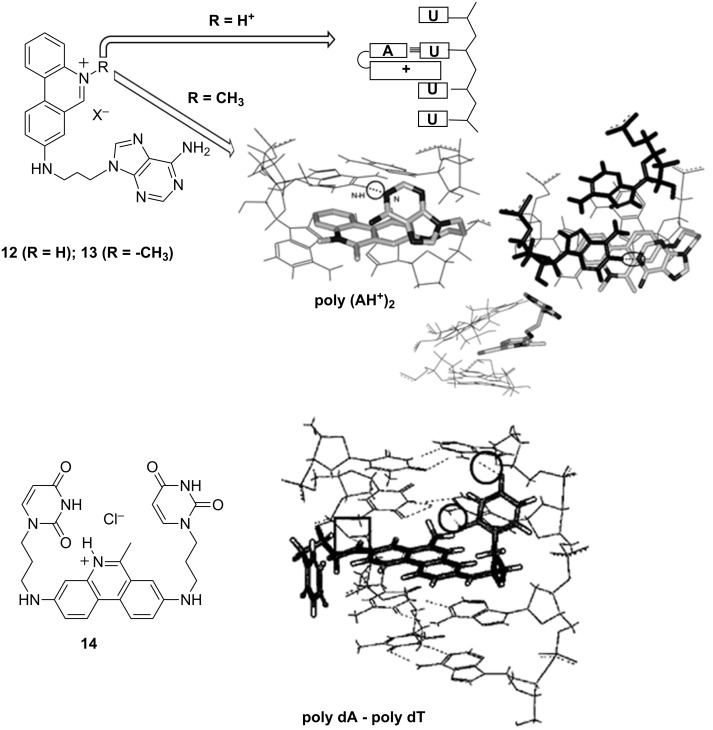
Top: Recognition of poly(U) by **12** and ds-polyAH^+^ by **13**; bottom: Recognition of poly(dA)–poly(dT) by **14**, intramolecular H-bonds marked by circles. Reproduced with permission from [72–73], copyright 2002, 2005 The Royal Society of Chemistry and with permission from [71], copyright 2003 John Wiley & Sons, Inc.

In an effort to influence DNA sequence-selective recognition by small molecules (MW <1000), our group prepared a series of phenanthridine derivatives with one or two nucleobases covalently attached at the 3 and/or 8 positions of the phenanthridine ring (Scheme 23). The phenanthridinium–nucleobase conjugates did not show targeted selectivity toward complementary nucleotides in aqueous medium due to the strong competition of bulk water with the expected hydrogen bonds [70–71]. Fortuitously, the hydrophobic environment within the common DNA/RNA binding sites allowed H-bonding-based recognition of some complementary polynucleotide sequences. However, the recognition pattern was not straight-forward; for instance N5-protonated phenanthridinium–adenine derivative **12** successfully recognized a complementary poly(U) sequence [72] (Scheme 23), but this recognition was completely lost upon introduction of a permanent positive charge by methylation of phenanthridine-N5 **13** [71]. Intriguingly, N5-methylated phenanthridine–adenine conjugate **13** exhibited preferred binding to peculiar protonated poly AH^+^ double stranded helix (Scheme 23) [71]. Attachment of two adenines to N5-protonated phenanthridinium completely abolished interactions with DNA and RNA due an extensively self-stacked structure but the bisuracil–phenanthridinium conjugate **14** was able to distinguish between alternating and consecutive AT sequences by peculiar combination of aromatic stacking and hydrogen-bonding interactions [73–74].

**Figure 6:**
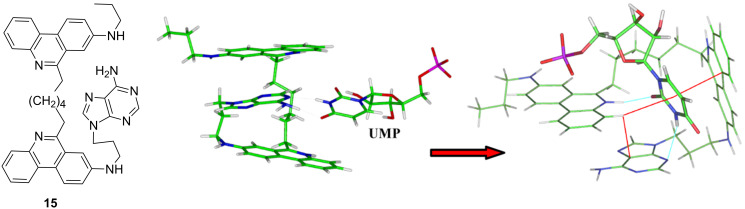
The bis-phenanthridinium–adenine derivative **15** (LEFT) showed selectivity towards complementary UMP; structure of the **15**–UMP complex (RIGHT) obtained by molecular modelling. Reproduced with permission from [75]. Copyright 2010 Elsevier Ltd.

At variance to phenanthridinium–nucleobase conjugates (Scheme 23), which were not able to differentiate among mononucleotides, some bis-phenanthridinium–nucleobase conjugates provided a more convenient binding site for the nucleobase. For instance, adenine derivative **15** (Figure 6) selectively recognized the complementary nucleotide (UMP) by specific change in the UV–vis spectrum of phenanthridine subunits and high affinity [75]. Molecular modelling studies proposed a structure of the **15**–UMP complex stabilized by a set of intra- and intermolecular stacking interactions and intermolecular hydrogen bonds unique for derivative **15** interaction with UMP but not possible with other nucleotides. Moreover, mentioned bis-phenanthridinium–nucleobase conjugates also exhibited complex interactions with various ds- and ss-DNA and ds- and ss-RNA, whereby the thermal denaturation and ICD signal-based sensing was highly sensitive to the polynucleotide basepair composition and secondary structure [76]. However, the low solubility of the studied systems hampered NMR studies and the very complex set of possible interactions did not allow accurate structural explanation of observed ICD recognition.

Laborious synthetic procedures for the preparation of bis-phenanthridine–nucleobase conjugates initiated a novel, convergent and much more flexible approach relying on solid phase peptide synthesis described earlier (Figure 4). In such a manner prepared phenanthridine–thymine conjugates [77], intercalated into ds-DNA whereby binding was marginally influenced by attached thymine and the peptide backbone. More intriguing was the observed excimer fluorescence emission and the very specific CD spectrum of pentapeptide confirming the very efficient phenanthridine–thymine–phenanthridine stacking. The obtained results support efficient and predictable self-organisation of sterically crowded oligo-phenanthridine peptides (Figure 4, [68]) as well as analogues containing other (DNA and RNA binding) aromatic moieties [77], which as a proof of principle support future design of analogous peptide libraries for combinatorial approach to recognition of various DNA and RNA targets.

**Figure 7:**
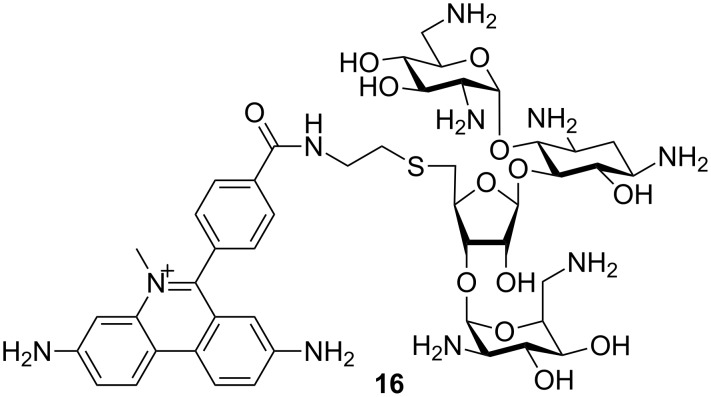
The neomycin–methidium conjugate targeting DNA:RNA hybrid structures [80].

A structure–activity search revealed several phenanthridinium derivatives as promising binders to DNA:RNA hybrid structures [78]. Based on their previous work [79], Arya and coworkers designed neomycin–methidium conjugate **16** (Figure 7) [80], which selectively recognized the DNA:RNA hybrid duplex (poly(dA):poly(rU)) with sub-nanomolar affinity, much higher than the affinities shown for traditional aminoglycoside–nucleic acid targets. This joins the mentioned EB analogue to a small number of ligands that bind DNA:RNA hybrid structures. Latter play crucial roles in a number of biological processes (transcription, reverse transcription [79], the priming of DNA prior to replication [81], participating in different types of enzymatic activity, notably telomerases [82] and HIV RNase).

**Figure 8:**
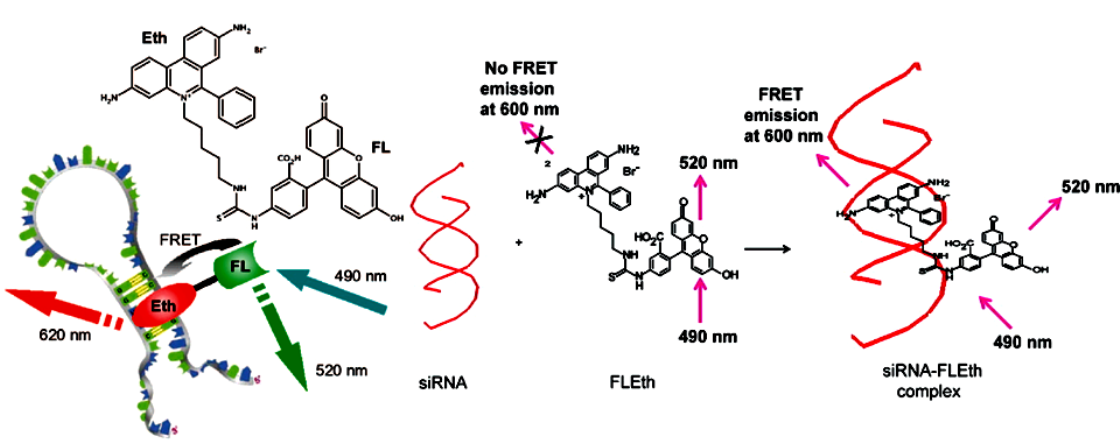
Two-colour RNA intercalating probe for cell imaging applications: Left: Chemical structure of EB-fluorescein conjugate (**FLEth**) and cartoon depicting the energy transfer process from fluorescein to the intercalated phenanthridine fluorophore. Reproduced with permission from [83]. Copyright 2008 American Chemical Society.; Right: Convenient Reporter for Small Interfering RNAs fluorophore. Reproduced with permission from [84]. Copyright 2009 American Chemical Society.

Ethidium bromide was introduced as a part of a heterogenic two-chromophore system, to take advantage of very efficient FRET energy transfer process (77%) from fluorescein to the RNA-intercalated phenanthridinium fluorophore (Figure 8, left) [83]. The resulting fluorescent dye exhibited improved ds-RNA-marker properties in comparison to other phenanthridinium analogues by means of signal brightness, signal-to-background noise and increased fluorescence half-lifetime. The same dye was also applied as convenient reporter for si-RNA (Figure 8, right) [84]. In parallel the designed and tested covalently linked ethidium bromide–ruthenium(II) complex also proved to be an imaging probe whose fluorescence intensity and lifetime changes substantially in the presence of RNA [85], thus supporting a strategy of phenanthridinium incorporation into the heterogenic two-chromophore system.

Phenanthridines are rarely combined with moieties covalently interacting with DNA and RNA. One of the most promising examples reported recently revealed that in a series of mono functional, cationic platinum(II) compounds, phenanthriplatin displayed a greater cytotoxic activity than either cisplatin or oxaliplatin despite a fact that binding to DNA induces a little distortion in the double helix (covalent adducts with DNA) [86]. The increased activity was attributed to improved cellular uptake and consequent inhibition of the cellular life cycle, whereby inhibition was additionally correlated to more expedient binding to nucleobases (5'-dGMP) in respect to less efficient binding of sulfur-containing nucleophiles present in resistance processes within the cell.

### Phenanthridine covalently bound to DNA and RNA

The phenanthridine aromatic moiety curvature nicely fits the shape of an average DNA and RNA basepair, while the length allows the incorporation of considerably long substituents at 3,8- positions available for attachment to DNA and RNA and/or various additional non-covalent interactions with the polynucleotide backbone.

**Figure 9:**
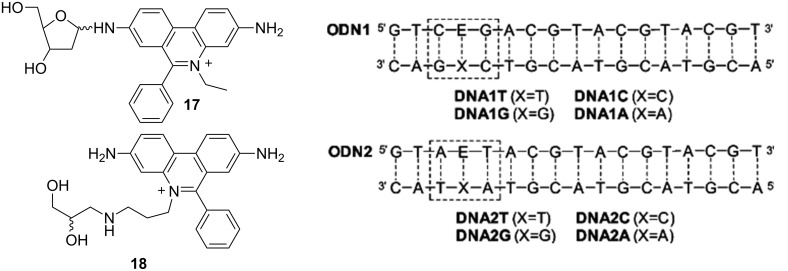
The ethidium bromide nucleosides **17** (top) and **18** (bottom). DNA duplex set 1 and 2 (E = phenanthridinium intercalation site). Reproduced with permission from [87]. Copyright 2004 American Chemical Society.

The ethidium bromide incorporated as an artificial DNA base (**18**, Figure 9) at specific sites in duplex DNA was used to study photoinducible charge transfer processes [87]. Upon attachment to the DNA chain the phenanthridinium base (E, Figure 9) was efficiently intercalated into the DNA oligonucleotide, not disturbing the position of adjacent basepairs nor the complementary oligonucleotide strand (abasic site X). Though, ethidium 2’-deoxyribofuranoside (**17**) [88] revealed chemical instability and was therefore replaced with an acyclic linker system [87]. However, in a later work the acyclic linker was again modified to correspond by length to the deoxyribofuranoside, whereby it was proven that structural changes do not influence significantly the EB insertion into the double helix, nor EB spectroscopic properties [87,89].

**Figure 10:**
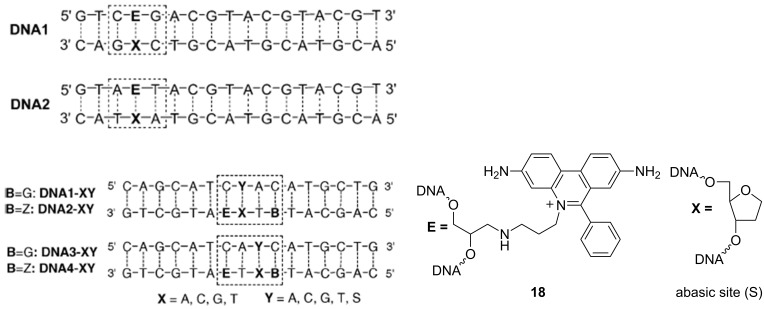
Left: various DNA duplexes; DNA1 and DNA2 used to study the impact on the adjacent basepair type on the EB fluorescence (reproduced with permission from [90], copyright 2004 John Wiley & Sons, Inc.) and **DNA1,2,3,4-XY** studying the EB fluorescence quenching by 7-deazaguanine (B) as a function of different position of abasic site (S). Reproduced with permission from [91] Copyright2005 Royal Society of Chemistry. Right: structure of incorporated EB (**18**) and of the abasic site (S).

Further studies revealed that various adjacent base pairs (Figure 10, A–T in DNA1, G–C in DNA2) did not significantly influence the spectroscopic properties of the ethidium bromide [90], while usage of noncovalently bound electron-acceptor showed applicability of the phenanthridinium–DNA system for studies of electron transfer in DNA [90]. Thus the EB-nucleobase fluorescence was not sensitive to the type of naturally-occurring adjacent basepairs [90] but showed to be sensitive to major erroneous ds-DNA sites (e.g., abasic sites) [91]. Namely, by using the well-known system of EB-fluorescence quenching by 7-deazaguanine incorporated within modified oligonucleotides, it showed that the abasic site (S) either one base pair away (**DNA1-XY** and **DNA2-XY**) or two base pairs away (**DNA3-XY** and **DNA4-XY**) from the EB chromophore showed an enhanced fluorescence quenching compared to the matched duplexes [91].

Among many studies of charge transfer in DNA, several applying ethidium bromide, revealed an unexpected complexity of the process, pointing out the importance of the DNA/EB complex flexibility on the efficiency of the transfer. A study of comparatively flexible DNA/EB complex, EB covalently attached to the 5’-end of oligonucleotides, in detail described the rate and distance dependencies of charge transfer through DNA [92–93]. A more rigid type of EB-binding, whereby the EB-nucleobase was incorporated close to the centre of the DNA oligomer in combination with two different charge acceptors (7-deazaguanine as an electron hole acceptor and a 5-nitroindole as a suitable electron acceptor) [94], showed similar rates and distance dependencies for both, electron and hole transfer. The obtained results [92–94] stress the importance of DNA-basepair dynamics for the electronic transfer processes in DNA-stacks. The efficiency of transfer is rather more controlled by motions of chromophores involved in aromatic stacking of DNA-reporter complex than with rigid aryl-stacking, thus suggesting the presence of a base gating mechanism (for the here presented EB/DNA systems on the 10–100 ps scale).

### Phenanthridine analogues

One of the main incentives that increased interest in phenanthridines was the large family of naturally occurring close analogues, mostly of extended aromatic moieties (e.g., benzo-phenanthridines). Their distinctive biomedical properties resulted in a considerable amount of research and large number of publications, hampering their detailed description in this review. Nevertheless, several chosen examples of phenanthridine analogues will be presented.

**Figure 11:**
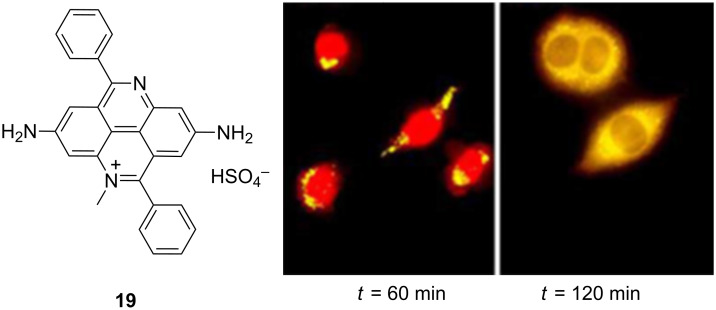
Structure of 4,9-DAP derivative **19**; Rright: MIAPaCa-2 cells stained with 10 μM **19** after 60 and 120 min incubation, respectively. Magnification 630×. Reproduced with permission from [95]. Copyright 2000 Royal Society of Chemistry.

The phenanthridine analogues, 4,9-diazapyrenium cations (very scarcely studied) [95], revealed a number of very intriguing properties upon binding to DNA and RNA. For instance, the closest analogue to ethidium bromide **19** (Figure 11) showed opposite fluorescence response upon binding to double-stranded GC-DNA and GC-RNA (quenching of emission) and AU(T) (emission increase) [96]. The only plausible structure of intercalated **19** requires parallel positioning of **19** and adjacent base pairs’ long axes, consequently positioning the bulky phenyl substituents of **19** in opposite DNA grooves – thus **19** exhibits rare threading intercalation binding into double-stranded polynucleotides. Furthermore, derivative **19** formed two types of complexes with ss-RNA, a more stable one with a well organised, possibly helical structure (ICD evidence) close to saturation of poly(U) (*r* ≈ 1) and less stable complexes with the other ss-RNA, characterised by decreased CD bands of polynucleotides. At variance to other 4,9-diazapyrenium compounds that lack the amino groups in positions 2 and 7, derivative **19** exhibited higher affinities and larger stabilisations of ds-DNA and ds-RNA probably due to additive interactions of its amino substituents within the polynucleotide binding site. All 4,9-DAP derivatives also showed considerable antiproliferative activity, interestingly only **19** having strong, micromolar activity in vitro but negligible in vivo toxic effects in mice [97]. Strong fluorescence of **19** allowed monitoring of the very efficient cellular uptake (Figure 11), upon which red colour of **19** accumulated in cell nuclei – intriguingly after only 2 hours fluorescence colour changed to yellow (Figure 11, right) and the dye distributed over the cytoplasm pointing out to the metabolic modification of the compound.

The new, easily accessible analogue, dihydroimidazophenanthridinium cation characterised by cyclic structure connecting positions 5 and 6, showed promising antiproliferative activity [3,98–99]. Molecular modelling results and some preliminary experiments suggest intercalative binding mode, however up till now interactions with various DNA and RNA were not studied in detail.

**Figure 12:**
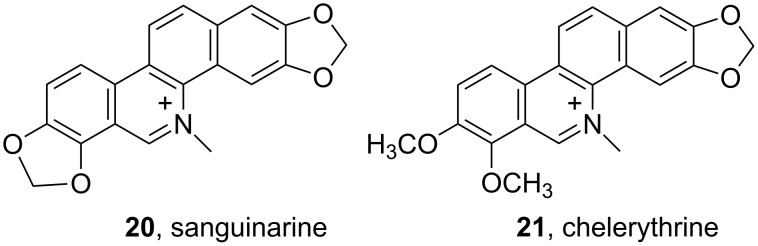
Examples of naturally occurring phenanthridine analogues.

One of several reasons for the increased research on phenanthridines is the discovery of naturally occurring analogues, e.g., some protoberberine alkaloids (Figure 12, sanguinarine and chelerythrine), widely distributed in several botanical families exhibiting many therapeutic applications. Very extensive results would require a focused review, thus some examples are listed below as outline of the importance.

Most of the sanguinarine (**20**) and chelerythrine (**21**) derivatives were typical DNA and RNA intercalators [100], some of them showing also intriguing interactions with ss-RNA, poly(A) [46]. However, either intercalative binding mode or structural similarity to EB did not hamper their biomedical applications. For instance, in a series of 5-methylbenzo[*c*]phenanthridinium derivatives, based on combination of sanguinarine (**20**)/chelerythrine (**21**) structures [101], the presence of a 1-phenyl or 12-phenyl substituent on 2,3,8,9-tetramethoxy-5-ethylbenzo[*c*]phenanthridinium chloride [102] significantly enhances the antibacterial (*Staphylococcus aureus* and *Enterococcus faecalis*) activity relative to sanguinarine. Another example, using the strategy of bioactivity-guided fractionation, the bioactive compound chelerythrine (**21**, a quaternary benzo[*c*]phenanthridine alkaloid) was isolated from *Chelidonium majus L.* [103]. In addition to strong antihelmintic activity (against D. intermedius), chelerythrine also showed antimicrobial, antifungal and anti-inflammatory activity [104].

### Discussion of the presented results and perspectives

The data presented in this review endeavoured to stress the outstanding properties of the rather simple and, due to the substantial advance in synthetic approaches, now readily available moiety (phenanthridine). Within the last 15 years significant research efforts invested in the phenanthridine and phenanthridinium structure–DNA and RNA-binding relations resulted in a significantly advanced understanding of the chromophore system in the free form and even more important in complex with ds-DNA and ds-RNA.

Detailed analysis of the DNA and RNA binding parameters (Table 1) revealed that contrary to the common paradigm about ethidium bromide derivatives as classical ds-DNA and ds-RNA intercalators, here presented results show a large variety of binding modes, very often the same molecule exhibiting more than one binding mode, depending on the ratio *r* = [compound]/[polynucleotide]. Moreover, there is no set of rules which will accurately predict the dominant binding site of newly designed phenanthridine/phenanthridinium analogues. All aforementioned also hampers the prediction of the fluorimetric response, which is much more dependent on the binding mode than on the substituents attached to the chromophore. However, by the rule of thumb, if phenanthridine substituents at 3,8-positions sterically allow the intercalation into ds-DNA or ds-RNA, than a binding affinity within the micromolar range could be expected and the systematic research of Luedtke et al. [48] allows predictions of the fluorimetric response, while results of Wagenknecht et al. [88–94] as well as Turro et al. [83–85] are applicable for the design of DNA charge transfer processes.

**Table 1:** Binding affinities (log *Ks*); thermal denaturation effects and proposed binding modes of chosen phenanthridine and phenanthridinium derivatives with natural and synthetic polynucleotides: ds-DNA and ds-RNA.

Ligand	pH	log *K*_s_^a^(*∆T_m_*^b^) / Binding mode^c^					
		ctDNA	dA–dT	dAdT–dAdT	rA–rU	dG–dC	rG–rC

**1**	7.5	5.6^d^ ,(–) / IC	–	–	–		–
	
**4**	5	–, (20.6) / MG	7.4, (29.5) / MG	–, (25.0) / MG	7.4, (35.4^e^/–1.8) / IC	6.6, (–) / IC	–
	7	–, (5.6) / MG	6.5, (9.8) / MG	–, (10.3) / MG	5.7, (5.0) / NSA	5.8, (–) / IC	
	
**5** R = (CH_2_)_4_	6.2	–	5.2, (–^f^/45.3) / IC + ESI	–	5.7, (–^f^/21.5) / IC + ESI	–	6.5,(–) / IC + ESI
	
**5** R = (CH_2_)_6_	6.2	–	5.7, (–^f^/42.5) / IC, NSA	–	8.0, (–^f^/20.7) / IC, NSA	–	6.3,(–) / IC, NSA
	
**6** R = (CH_2_)_6_	6.2	–	–^g^, (25.6) / GB	–	5.3, (3.9) / GB	–	6.5,(–) / GB
	
**7**	6.2	–	6.0, (–) / GB, ESI	–	–^g^ ,(–) / GB, ESI	–	5.7,(–) / GB, ESI
	
**8**	5	5.8, (25.8) / IC, ESI	–, (11.1) / IC, ESI	–	5.1, (9.5) / IC, ESI	–	5.8,(–) / IC, ESI
	7	6.6, (5.7) / IC	–, (12.1) / IC	–	6.2, (5.2) / IC	–	5.2, (–) / IC
	
**9** (*n* = 2)	5	5.5, (2^f^/22.6) / MG	5.8 (3.3^f^/27.7) / MG	–	5.2 (0^e^/1.1) / NSA	–	6.6^h^, (–) / NSA
	
**9** (*n* = 6)	5	6.3, (2.9) / MG	5.7, (3.4) / MG	–	5.2 (0^e^/0.9) / NSA	–	6.8^h^, (–) / NSA
	
**9** (*n* = 9)	5	6.0, (7.7) / IC	6.1, (3.8) / IC	–	6.0, (10.6^e^/–2.0) / IC	–	5.0, (–) / IC
	
**10** R = Gly	5	5.,5 (8.1) / IC	–	–	–	–	–
	7	4.7, (–) / IC	–	–	–	–	–
	
**11** R = Gly	5	6.9, (16.5) / IC	–	–	–	–	–
	7	6.0, (–) / IC	–	–	–	–	–
	
**14** (bis-uracil analogue)	5	–	4.6, (18.0) / PIC, HB	5.1 / PIC	5.0, (2^e^/12.4) / PIC	–	–
	
**14** (bis-adenine analogue)	5	–	5.0, (6.0) / MG	5.3, (1.1) / MG	5.1, (1.5^e^/0.3) / GB	–	–
	
**16**	5.5	–	–, (7.7^i^) / IC, MB	6, (7.4^i^ ) / IC, MB	9.3, (34.3 ^i^) / IC, MB	–	–
	
**19**	5	6.4, (19.6) / TIC	5.4, (10^f^/20) / TIC, MB	6.1 / TIC	6.5, (35.6) / TIC	6.1, (–) / TIC	6.8, (–) / TIC
	7	–, (19.4) / TIC	5.6, (11.3^f^/21.6) / TIC, MB	5.9 / TIC	6.5, (25.5) / TIC	6.9^j^, (–) / TIC	6.1, (–) / TIC
	
**20**	6.5	–	–	–	5.9, (19.0^k^) / IC	–	5.5,(–) / IC

^a^Binding constants calculated from titration data by processing according to the Scatchard equation. ^b^r = [compound]/[polynucleotide] = 0.2 if not stated otherwise. ^c^IC = intercalation; MG = minor groove; NSA = non-specific agglomeration; GB = undefined groove binding; BIC = bis-intercalation; ESI = electrostatic interaction; HB = hydrogen bonding; PIC = partial intercalation; MB = mixed binding mode; TIC = threading intercalation. ^d^Binding constants calculated from ethidium bromide displacement experiments [52]. ^e^Biphasic thermal denaturation transitions at pH 5 due to different RNA forms [62]. ^f^Biphasic thermal denaturation transitions due to ^b^MB, values for both transitions given when possible. ^g^Not possible to calculate due to systematic deviation of experimental data from best-fitted Scatchard isotherm. ^h^Cumulative binding constants for mixed binding mode. ^i^r = [compound]/[polynucleotide] = 0.1. ^j^Polynucleotide is poly(dG–dC)_2_. ^k^r = [compound]/[polynucleotide] = 0.5.

However, these rules do not apply for interactions of phenanthridine/phenanthridinium derivatives with significantly more flexible single stranded (ss-)polynucleotides, for instance ss-RNA (Table 2). The data about interactions with ss-DNA or ss-RNA are sparse and deficient, mostly determined for derivatives with substituents aiming toward particular nucleobase recognition, with very few referent compounds for any final conclusion about the binding properties of phenanthridine moiety alone. Nevertheless, binding data obtained for ethidium bromide and 8-amino-substituted derivatives with methylated or protonated heterocyclic N5 (Table 2) show that the phenanthridine/phenanthridinium cation interacts with purine ss-sequences with affinity approximately one–two orders of magnitude lower in comparison to ds-DNA or ds-RNA, while interaction with pyrimidine ss-polynucleotides is even one order of magnitude lower. This agrees well with the aromatic stacking interactions between phenanthridine and nucleobase as dominant binding interaction (most likely intercalation), while differences between permanent (EB, PHEN-Me) and reversible (PHEN-H^+^) positive charge do not play a significant role. Intriguingly, EB revealed an order of magnitude lower affinity toward poly(A) in comparison to PHEN-Me and PHEN-H^+^, which could be attributed to the steric hindrance of EB at C6 and N5 positions to the optimal orientation of phenanthridinium within the intercalative binding site between adjacent nucleobases. As expected, bis-phenanthridine derivatives exhibited higher affinity due to the bis-intercalative binding mode, and in some cases show a fluorimetric recognition of a particular ss-polynucleotide (e.g., **8**) due to the fine interplay of binding interactions. Again, very scarce information about the complex structure did not allow accurate determination of binding contributions, which would clarify the observed selectivity.

**Table 2:** Binding affinities (log *Ks*) and proposed binding modes of chosen phenanthridine and phenanthridinium derivatives with synthetic polynucleotide ss-RNA.

ligand	pH	log K_s_^a^ / Binding mode^b^				
		pA	pU	pG	pC	pA^c^+ (∆T_m_)^d^

PHEN-Me^e^	5 7	4.3 / IC 4.8 / IC	<3 / IC <3	–	–	
	
PHEN-H^+f^	5	5.1 / IC	<3 / IC	–	–	
	
EB [96]	5 7	3.3 / IC 3.9 / IC	<3 / IC <3 / IC	3.8 / IC 3.1 / IC	3.3 / IC <3 / IC	
	
**5** R = (CH_2_)_4_	6.2	4.2 / IC, NSA	3.7 / IC, NSA	5.3 / IC, NSA	–	–
	
**5** R = (CH_2_)_6_	6.2	3.8 / IC, NSA	4.1 / IC, NSA	5.8 / IC, NSA	–	–
	
**6** R = (CH_2_)_6_	6.2	4.1 / IC, NSA	4.2 / IC, NSA	5.4 / IC, NSA	–	–
	
**7**	6.2	6.3 / BIC	5 / BIC	7.1 / BIC	–	–
	
**8**	5	5.0 / IC	4.5 / IC	6.1 / IC	5.4 / IC	–
	7	4.6 / IC	4.4 / IC	5.1 / IC	4.5 / IC	–
	
**12** (phenanthridinium-adenine)	5	5.3 / IC	4.5 / IC + HB	–	–	–
	
**12** (phenanthridinium-uracil)	5	5.3 / IC	>3^g^ / IC	–	–	–
	
**13**	5	–	>3 ^g^/ IC	–	–	5.3, (3) / IC, ESI
	7	–	>3^g^ / IC	–	–	4.4 / IC
	
**14** (bis-uracil analogue)	5	–	–	–	–	5.2, (8.0) / PIC, HB
	
**14** (bis-adenine analogue)	5	–	–	–	–	5.1, (0.5) / MG
	
**19**	5	–	3.9 / IC	4.7 / IC	2.9 / IC	–
	7	4.1 / IC	4.4 / IC	4.5 / IC	2.6 / IC	–
	
**20**	6.5	–	–	–	–	4.5, (6.0) / IC

^a^Titration data were processed according to the Scatchard equation. ^b^IC = intercalation; MG = minor groove; NSA = non-specific agglomeration; BIC = bis-intercalation; ESI = electrostatic interaction; HB = hydrogen bonding; PIC = partial intercalation. ^c^poly A at pH 5 is mostly protonated and forms ds-polynucleotide [62]. ^d^r = [compound]/[polynucleotide] = 0.2, only for **20** r = 0.5. ^e^8-(Propylamino)-5,6-dimethylphenanthridinium cation [71]. ^f^8-(Propylamino)-6-methylphenanthridine [70]. ^g^Estimated value due to less than 20% of complex formed.

Although only the current widespread biochemical application is focused on ethidium bromide/propidium iodide dyes for DNA dyeing and cell viability tests, results summarised in this review pointed out the intriguing potential of the phenanthridine/phenanthridinium system for chemical and biochemical research. Widely used fluorimetric dyes, such as cyanine derivatives, are non-fluorescent in the free state but give tremendous fluorescence emission upon binding to biomacromolecular targets. However, many of these dyes show photobleaching, a significant overlap of the absorption and emission spectrum (minor Stokes shift) and the chemical stability in stock solution is often declared by the producer to last only several months. Although the phenanthridine/phenanthridinium system in principle does not show the ideal combination of non-emissive form in the free state/very strong emission in the bound state, it has several advantages over cyanine dyes: phenanthridine/phenanthridinium fluorescence is characterised by a large Stokes shift (up to 100 nm) allowing the full use of absorption maxima as well as easy incorporation in FRET systems, high resistance to photobleaching and mostly very high chemical stability. Biomedical use in human medicine was deterred by the potential carcinogenic and mutagenic properties of some derivatives (EB and analogues) but this is recently reassessed due to the evidently innoxious treatment of African trypanosomiasis in livestock for more than 40 years (isometamidium chloride hydrochloride and ethidium bromide [105]), together with recent results on phenanthridine-based alkaloids and the promising bioactivity of phenanthriplatin [86].

All aforementioned gave the impetus to the phenanthridine/phenanthridinium system research, which made significant progress in the study of the most common phenanthridine substituent positions (3-, 5-, 6-, 8-). Nevertheless, there are still many promising targets, for instance systematic study of various substituents attached at rarely used positions (1-,2-,4-,7-,9-) would be of high interest, especially since natural phenanthridine alkaloids (Figure 12) are richly substituted on these positions and very likely owe a lot of biological activity to particular type of substituent. Several other phenanthridine characteristics such as reversible positive charge introduction by protonation of the heterocyclic nitrogen (N5) were for the first time applied in designed DNA and RNA interactions, offering new biomedical applications – for instance, taking advantage of the significantly lower extracellular pH of many solid tumors [106], to which some antitumor drugs base their preferential accumulation in tumor tissue due to the weakly acidic p*Ka* value [107]. Furthermore, phenanthridine was very scarcely used as a ligand in metal coordination chemistry of biomedically oriented research, although heterocyclic nitrogen (N5) and/or various side-arm substituents offer many possibilities – as for example, very recently reported recognition of nucleotides by phenanthridine–lanthanide conjugates [108]. Finally, there are almost unlimited possibilities of phenanthridine incorporation into heterogenic fluorescent probes, taking advantage of the aforesaid phenanthridine spectrophotometric characteristics.
